# Supplementary material for: The discourse of civic pride: Hawleri identity as one of the oldest Kurdistani cities in the Middle East
Source: PLoS One. 2021 Nov 17;16(11):e0259231. doi: 10.1371/journal.pone.0259231 (PMC8598024; doi:10.1371/journal.pone.0259231)
Supplement: Attachment — Submitted filename: Architectural identity.docx [file pone.0259231.s001.docx]

The issue of architectural identity has become a common topic in architectural debates. We think we need more studies of the federal region identity

Based on literatures from social science and humanities, this inquiry of “what is the identity” leads one to a question of personality (who are you?). The question of identity is often interpreted to be a question about people's concepts of “who they are” and how they relate to others…please apply both moral and physical factors on the issue of identity.

In term of statistical analysis, we think that the study need more improvement by adding Factor analysis, multi regression analysis or correlation between study factors…. depending on descriptive analysis will affect the originality of the study…
