# Supplementary material for: The discourse of civic pride: Hawleri identity as one of the oldest Kurdistani cities in the Middle East
Source: PLoS One. 2021 Nov 17;16(11):e0259231. doi: 10.1371/journal.pone.0259231 (PMC8598024; doi:10.1371/journal.pone.0259231)
Supplement: Attachment — Submitted filename: Reviewer comments I.docx [file pone.0259231.s002.docx]

**Answer to the Reviewer’s comments and description of the changes introduced in the revised version of manuscript PONE-D-20-20350, submitted to the Journal of PLOS ONE**

# Editor's comments:

Dear Dr. Sherwani,

Thank you for submitting your manuscript entitled "The Discourse of Civic Pride: Hawleri Identity as one of the oldest Kurdistani cities in the Middle East" to PLOS ONE. Your manuscript files have been checked in-house but before we can proceed we need you to address the following issues:

1. Thank you for your reply. Please note that the requirement for informed consent is distinct from the requirement for ethical approval. Participants should be informed of and agree to participation in research, unless data is analyzed anonymously.

Please see the guidance below:

Please provide additional details regarding participant consent. In the Methods section, please ensure that you have specified (1) whether consent was informed and (2) what type you obtained (for instance, written or verbal). If your study included minors, state whether you obtained consent from parents or guardians. If the need for consent was waived by the ethics committee, please include this information.

This study involved (human participants) and was reviewed and approved by the ethics committee of the Media Techniques Department, Erbil Technical Administrative College, Erbil Polytechnic University, Erbil, Iraq. With respect to the ethical consideration, the author assured the respondents with the cover letter about the anonymity with respect to their responses. Moreover, the author followed all ethical measure to complete the research, section 3 has discussed some detailed procedure. To answer the questions asked for the objective of this study, a large amount of data is gathered through the city social media channels and randomly distributing a survey among a large number of people from Erbil. For both groups, the questions were given after they expressed their willingness to participate as respondents of this research. Then, through a telephone call to inquire about the availability of all, before proceeding to send a survey through email. Due to respondent’s comfortability and upon their request we obtained only verbal consents because they were not willing to provide written approval.

2. Thank you for letting us know the DOIs to access the data were included in your references section.

Thank you very much for your comment. The DOI was added for all references. In the case that the DOI was not exist, the published link was added.

Note: the last reference was just accepted. That is why it does not have DOI. Nevertheless, we added the acceptance latter at the end of this file. In addition, we added the accepted paper based on the guidance given by the journal (it is allowed by the journal)

# Reviewer #1:

Reviewer #1:

First of all, the author appreciates the overall evaluation of the paper, as well as the thorough review and valuable suggestions.

1) The writing is fine for the most part, but author needs to watch for typos and writing mistakes more. Too many appear for a work under review for publication.

| Reviewer Comment | 1 |
| --- | --- |
| Authors’ response | The author agrees with the reviewer’s remark. After an extensive review, indeed, we have found several typos and technical errors.  Accordingly, the following changes have been made in the new manuscript. |
| Notes/action | Please, see the changes in the following sections:  For example,  **Abstract**  For that purpose, a critical discourse analysis approach was used to study the city identity of “Hawleri” people of Erbil city which is the capital and the most populated city in the Iraqi  **1^st^ paragraph in section 1:**  Studying the identity of urban people is not easy due to the fact that apart from identity and discourse, this kind of study interferes with a lot of other disciplines such as sociology, politics, psychology, and research methodology in urban studies.  **3^rd^ paragraph in section 1:**  In fact, these rivalries and competition between the cities may attract the attention of political communities and local governments.  **6th paragraph in section 1:**  Hawleri is a local term used by Kurdish people to refer to people from Erbil which is the capital city of the Iraqi Kurdistan Region. Hawler is the local name of the city and “*i*” is a suffix that indicates the people of a certain place in the Kurdish language.  **7th paragraph in section 1:**  Finally, it is important to show what is the novelty of this study and what is the reason to choose the oldest cities in the Middle East as a case study (i-iv).  **1^st^ paragraph in section**  Some other references focused on civic pride and community identity and how people from different places show their belongings [5, 15].  As mentioned in the introduction section, in terms of methodology and dataset, this study is different from the previous studies showed above due to the fact that the critical discourse analysis was used to reveal the civic pride and people’s identity of a city in an unidentified region.  searches for the constituents of human existence, among other things. These basic features of all humans he calls “existentialia”,  pride is a value that tends to generate certain life up to ideals or expectations.  **2^nd^ paragraph in section 2**  Southworth [17] reported that urban identity is the best-recognized parameter in representation beyond time and includes a deep understanding  **3^rd^ paragraph in section 2**  Since the scope of the urban identity aspects is very wide, Figure 1 is drawn in order to simplify the information given in the literature. Accordingly, the urban identity aspects can be classified into three distinct features  **Last paragraph in section 2**  Socially and Sociolinguisitcally, Hawleri is not a dialect by itself; it is part of a larger dialect that is spoken in a larger geographical region of Southern and Eastern Kurdistan.  **1^st^ paragraph in section 3**  According to the main concept of the selected approach, the questions (Q1-5) showed in Figure 2 are asked by Halwrei people.  **1^st^ paragraph in section 4.1**  The name #Lo (لؤ#) is a word that means “why” in some contexts and “to a” in  **3rd paragraph in section 4.1**  Erbil has an amazing continuity of its name (Irbilum, Urbilum, Urbel, Arbail, Arbira, Arbela and Erbil/Arbil)  **1^st^ paragraph in section 4.2**  The following two tables (Tables 1&2) show the results of the survey. The survey is divided into two parts.  **3^rd^ paragraph in section 4.2**  born outside the Kurdistan region. Here, the data shows that the majority of the respondents are either born in the center of Erbil town or they live there.  Table 1 -The demographic profile of the study’s respondents  MA, MSc. and Ph.D. Holders  **2^nd^ paragraph in section 4.2**  The demographic information shows that people of different ages have participated, 2.4%  **7^th^ paragraph in section 4.2**  Here, according to the feedbacks and beliefs of the majority of the participants, 54.3%, those who serve the city are regarded as Hawleris.  **10^th^ paragraph in section 4.2**  about 34.2% of participants said that they are proud of being a Hawleri because Hawleris have nice traditions  **12^th^ paragraph in section 4.2**  regarded as Hawleris and 22% of those who born outside Hawler but live in Hawler are regarded as Hawleris.  **Last paragraph in section 4.2**  The last two questions (Q17&18), are concerned with history. In historical terms, 86.5% stated that hundreds of years ago Erbil was a Kurdish city  **4^th^ paragraph in section 5**  Erbil was an Assyrian city thousands of years ago. At those times there were not national states and countries like Arabs, Kurds, Turks, etc.  **Last paragraph in section 5**  This attitude excludes many decent families who have lived in Erbil for many decades.  **1^st^ paragraph in section 6**  In this study, the discourse of civic pride and urban identity of the people are studied due to the fact that the knowledge on these two parameters is very scarce, |

2) The citation style here is one I’ve not seen before – is this the accepted reference style for this journal?

| Reviewer Comment | 2 |
| --- | --- |
| Authors’ response | The author agrees with the reviewer’s remark. The reference style was not the same as the one recommended by the journal. Based on the reviewer's remark, we followed the instruction of the journal (A reference management tool, EndNote, offers a current **style file** that can assist you with the formatting of your references. If you have problems with any reference management program, please contact the source company's technical support.)  **style file:**  <https://endnote.com/style_download/plos-public-library-of-science-all-journals/> |
| Notes/action | Since we used the Endnote software, it automatically updated the references according to the style of the journal. |

3) In the methodology section, author should probably comment a bit about how a question posed to a FB group does not result in a random sampling, since only those who choose to comment/answer the question, as well as join this group, have responses.

| Reviewer Comment | 3 |
| --- | --- |
| Authors’ response | The author thanks the reviewer for the remark. The author agrees to add the above statement.  Based on the reviewer's remark, the 2^nd^ paragraph in section 2 was altered. |
| Notes/action | Please, see the second paragraph in section 2.  To answer these questions, a large amount of data is gathered through the city social media channels (section 4.1), more specifically the Facebook group #Lo (لؤ# in Hawleri Kurdish) and randomly distributing a survey among a large number of people from Erbil (section 4.2). The data of the Facebook group are analyzed qualitatively and the survey responses are analyzed quantitatively. In order to avoid getting any random or unreliable sample (response), a strict boundary was considered to get the data. Thus, only those who answered all questions were considered for both the online community (i.e. those who joined the Facebook group #Lo) and people from the survey (i.e. academic people). Further details regarding the dataset and the way how they were collected, namely for the online community and people from the survey are shown in sections 4.1 and 4.2, respectively. In general, 236 participants in the social media and 573 participants in the survey properly answered the questions showed in Figure 2, respectively. In general, 236 participants in the social media and 573 participants in the survey properly answered the questions showed in Figure 2, respectively. |

4) On the issue of education of respondents, some more needs to be said. With most of the respondents having a university education, it seems to me that they are not representative of Erbil’s population in general (most of whom do not have a university education).

| Reviewer Comment | 4 |
| --- | --- |
| Authors’ response | As mentioned before, in order to avoid getting any random or unreliable sample (response), a strict boundary was considered to get the data. Thus, only those who answered all questions were considered from the online community and the survey. Unfortunately, we had to discard many responses due to the mentioned boundary which were came from non-academic people.  Nevertheless, we have to consider the fact that in discourse related matters (public options by leaders), many studies concluded that the leaders and academic people have a significant impact on ordinary people (public option). Thus, the author assumed that the output of this study may be representative.  According to the reviewer’s remark, we add a statement regarding the mentioned fact. Also, added few studies. |
| Notes/action | Please, see the second paragraph in section 3:  To answer these questions, a large amount of data is gathered through the city social media channels (section 4.1), more specifically the Facebook group #Lo (لؤ# in Hawleri Kurdish) and randomly distributing a survey among a large number of people from Erbil (section 4.2). The data of the Facebook group are analyzed qualitatively and the survey responses are analyzed quantitatively. **In order to avoid getting any random or unreliable sample (response), a strict boundary was considered to get the data. Thus, only those who answered all questions were considered from the online community (i.e. those who joined the Facebook group #Lo) and the survey (i.e. academic people). Further details regarding the dataset and the way how they were collected, namely for the online community and people from the survey are shown in sections 4.1 and 4.2, respectively. In general, 236 participants in the social media and 573 participants in the survey properly answered the questions showed in Figure 2, respectively. In addition, due to the strict boundary mentioned before, the percentage of people who concluded less than bachelor degree is low (~9%). In other words, most of the selected answers came from academic people because they answered all questions. Apart from the mentioned fact, this study can be still representative of Erbil’s population in general due to the fact that in discourse related matters (public options by leaders), many studies [****23-25****] concluded that the leaders and academic people have a significant impact on ordinary people (public option).**  New references:  23. Berger BK, Meng J. Public relations leaders as sensemakers: A global study of leadership in public relations and communication management: Routledge; 2014.  24. Mutz DC. Impersonal influence: Effects of representations of public opinion on political attitudes. Political Behavior. 1992;14(2):89-122.  25. Busemeyer MR, Garritzmann JL. Academic, vocational or general? An analysis of public opinion towards education policies with evidence from a new comparative survey. Journal of European Social Policy. 2017;27(4):373-86. |

5) P.22 – is it really justified from the data presented to say that Hawleris view themselves as “superior”?

| Reviewer Comment | 5 |
| --- | --- |
| Authors’ response | The author thanks the reviewer for the remark. Agreed, the mentioned word may be not adequate in the statement. Thus, the stamen has been altered according to the reviewer’s comment. |
| Notes/action | Please see, the last point in the conclusion section:  Hawleri Kurds sometimes exclude other nationalities and ethnic groups who live in the city. They are proud of themselves and they have only positive qualities and merits. |

6) (6a) The conclusion and analysis needs more. How does the author think that Hawleri identity competes with other forms of identity in Kurdistan? Does one feel Hawleri first and Kurdish second and a member of so and so tribe third and a Sunni (or Assyrian or something else) fourth, or some other order? Would a Kurdish Hawleri view a Turkmen Hawleri as closer to them than a Kurd from Suleimani? I know the data isn’t there to say much on the matter, but the author could speculate on such issues in the conclusion (“topics for further study….”). (6b) More analysis and explanation of the data itself is also needed in the main body of the paper.

| Reviewer Comment | 6 |
| --- | --- |
| Authors’ response | **6a - First part of the comment:**  Based on the reviewer’s comment, a new paragraph has been added at the end of the conclusion section.  **6b - second part of the comment:**  Indeed, the study required more analysis and correlation between the selected factors.  Apart from the discussion given in section 5, further analysis must be done in order to understand the effect of the Ethnic Backgrounds on the Hawleri identity and show a correlation between study factors. Thus, the responses of the participants have been classified based on their Ethnic Backgrounds. In addition, for each question, the most frequent response was selected and identified the percentage.  Based on the reviewer’s remark, several new paragraphs and new Table (Table 3 – see below) have been added to the manuscript in section 4-5. |
| Notes/action | 6a – Please, see the last paragraph in section 6  As a future study or to complement this work, the following questions can be considered for the survey: How does the author think that Hawleri identity competes with other forms of identity in Kurdistan? Does one feel Hawleri first and Kurdish second and a member of so and so tribe third and a Sunni (or Assyrian or something else) fourth, or some other order? Would a Kurdish Hawleri view a Turkmen Hawleri as closer to them than a Kurd from Suleimani?  See the last paragraph in section 4, and the last 5 paragraphs in section 5. |

**Finally**,

The authors believe all the comments of the reviewer were considered. We appreciate the fact that the quality of the paper has improved due to the valuable comments of the reviewer and we will gladly take any further suggestions from the reviewer into consideration.

# Reviewer #2

The topic is interesting. We think it will be so strong if the writer focused on Physical properties of architectural identity. The issue of architectural identity has become a common topic in architectural debates. Based on literature from social science and humanities, this inquiry of “what is the identity” leads one to a question of personality (who are you?). The question of identity is often interpreted to be a question about people's concepts of “who they are” and how they relate to others…please apply both moral and physical factors on the issue of identity.

| Reviewer Comment |  |
| --- | --- |
| Authors’ response | The authors appreciate the reviewer’s comprehensive comments, which further improved the quality of the paper.  The author agrees with the reviewer’s remark. The Physical properties of architectural identity is another parameter that must be considered. In other words, both moral and physical factors have a significant impact on identity matters. Nevertheless, the mentioned factors have been discussed by the same author in another paper that just have been accepted in the INCDTP journal (please, see the acceptance letter at the end of this file)  In order not to let the readers into error, a statement was added to the manuscript according to the reviewer’s remark.  Reference:  Sherwani K. The Discourse of Kurdish Traditional Textiles. Accepted for publication in the National Institute for Research and Development in Environmental Protection. 2021. |
| Notes/action | Please, see the last paragraph in section 4.2.  Apart from the selected factors, the Physical properties of architectural identity is another parameter that must be considered. In other words, both moral and physical factors have a significant impact on identity matters. Nevertheless, these parameters have not been discussed in this study due to the fact that the author of this study has been studied the mentioned factors in the previous study. |

In term of statistical analysis, we think that the study need more improvement by adding Factor analysis, multi regression analysis or correlation between study factors…. depending on descriptive analysis will affect the originality of the study…

| Reviewer Comment |  |
| --- | --- |
| Authors’ response | Indeed, the study required more analysis and correlation between the selected factors.  Apart from the discussion given in section 5, further analysis must be done in order to understand the effect of the Ethnic Backgrounds on the Hawleri identity and show a correlation between study factors. Thus, the responses of the participants have been classified based on their Ethnic Backgrounds. In addition, for each question, the most frequent response was selected and identified the percentage.  Based on the reviewer’s remark, several new paragraphs and new Table (**Table 3** – see below) have been added to the manuscript in sections 4-5. |
| Notes/action | Please, see one paragraph before the last paragraph in section 4.2:  Apart from the above facts, further analysis must be done in order to understand the effect of the Ethnic Backgrounds on the Hawleri identity and show a correlation between study factors. Thus, the responses of the participants have been classified based on their Ethnic Backgrounds (Table 3). In addition, for each question, the most frequent response was selected and identified the percentage. The output of this analysis has been shown in Section 5.  **See Table 3**  Also, the last 5 paragraphs in section 5.  According to the data shown in Table 3, the following facts can be discussed. The majority of Kurds and Christians believe that Hawleri is someone who serves Erbil, but Turkmens believes that Hawleri is the person who thinks he/she is from Erbil. Thus, Kurds and Christians judge based on the performance of the person while Turkmen cares about the way how the person believes (Table3, Q1).  Only 88% of Kurdish participants believe that they are Hawleri and proud of it. Whereas All the Turkmen and Christian participants believe that they are Hawleri and proud of it (Table3, Q2 and Q4). In addition, the majority of Kurds and Turkmen believe that they are Halwri because they are born in the city whereas Christians related the matter to the fact that they love Erbil and I am comfortable in the city (Table 3, Q3). In terms of the geographical, all Turkman participants believe that Hawleri is someone who Lived in Erbil Neighbourhoods (small area in the city center - inside the 60 Meter Road), and half of the Christian participants believe in the same matter except they slightly extended area (Inside the 120 Meter Road), whereas less than half of the Kurds believe that Hawleri is someone who Lived in Erbil region (bigger area - Erbil Governorate from Makhmoor to Haji Omeran) (Table 3, Q6).  In terms of birth, the ethnic background does not affect the response. The majority of them believe that Hawleri is someone whose ancestors were born in Erbil (Table 3, Q7). Nevertheless, in terms of the language they have different perspectives. The majority of Turkmen and Christians believe Hawleri is someone who speaks Kurdish and Turkmen whereas Kurdish participants believe Hawleri is someone who speaks Kurdish (Table 3, Q8). Furthermore, all the participants speak their language (Kurdish, Turkmani and other languages) at home (Table 3, Q10).  All Turkmen participants use the Hawleri dialect when talking to other people not from Erbil, whereas this percentage is less for Kurds and christen participants (Table 3, Q11). The same fact is true for Q12 (When I use the Hawleri Dialect with other people not from Erbil, I use "Lo). In terms of Q14 (clothes, Hawleri is someone who), Q15 (Which of the following characteristics are found in Hawleri) and Q16 (If you are given the opportunity to live, where would you stay?), the majority of the participants in all Ethnics are the same. In historical terms, None of the Ethnic (Turkmen, Christians, and Kurds) agree with each other. All of them believe that the city belongs to themselves (Table 3, Q17-18). |

**Finally,**

The authors believe all the comments of the reviewer were considered. We appreciate the fact that the quality of the paper has improved due to the valuable comments of the reviewer and we will gladly take any further suggestions from the reviewer into consideration.

*Table 3 - Responses of the Descriptive questions based on the ethnic Backgrounds (Kurd, Turkmen and Christians)*

| NO. | Descriptive questions | Ethnic Backgrounds | | | | | |
| --- | --- | --- | --- | --- | --- | --- | --- |
|  |  | **Turkmen** | % | **Christians** | % | **Kurd** | % |
| Q1 | Hawleri is someone: | Who thinks he/she is from Erbil | 56 | Who serves Erbil | 75 | Who serves Erbil | 54 |
| Q2 | Do you think you are Hawleri? | Yes | 100 | yes | 100 | Yes | 88 |
| Q3 | Why do you think you are Hawleri? | Because I was born in Erbil | 89 | I love Erbil and I am comfortable in the city | 75 | Because I was born in Erbil | 53 |
| Q4 | If you are Hawleri, are you proud of that? | Yes | 100 | Yes | 100 | Yes | 88 |
| Q5 | Why are you proud of being Hawleri? | Because Erbil residents have many nice customs | 56 | Because I don’t feel homesick in Erbil | 50 | Because Erbil has a glorious history | 39 |
| Q6 | In geographical terms, Hawleri is someone who... | Lived in Erbil Neighbourhoods (inside the 60 Meter Road) | 100 | Lived in Central Erbil (Inside the 120 Meter Road) | 50 | lives in Erbil Governorate from Makhmoor to Haji Omeran | 41 |
| Q7 | In terms of birth, Halweri is someone who … | Whose ancestors were born in Erbil | 89 | Whose ancestors were born in Erbil | 50 | Whose ancestors were born in Erbil | 47 |
| Q8 | In terms of language, Hawleri is someone who … | Speaks Kurdish and Turkmani | 67 | Speaks Kurdish and Turkmani | 50 | Speaks in Kurdish | 65 |
| Q9 | In terms of Dialects, Hawleri is someone who... | Speaks the dialect of Erbil | 56 | Speaks in Sorani - Slang Kurdish | 50 | Speaks the dialect of Erbil | 58 |
| Q10 | What languages do you speak at home? | Turkmani | 56 | Other languages | 100 | Kurdi | 97 |
| Q11 | Do you prefer to use the Hawleri dialect when talking to other people not from Erbil | Yes | 100 | Sometimes | 50 | Yes | 62 |
| Q12 | When I use the Hawleri Dialect with other people not from Erbil, I use "Lo | Feel proud of myself | 100 | I don’t know | 75 | Feel proud of myself | 80 |
| Q13 | In public ceremonies ... | I use the Hawleri Dialect | 56 | I use the Hawleri Dialect | 75 | I speak the slang Kurdish | 52 |
| Q14 | In terms of clothes, Hawleri is someone who | Wears Kurdish and formal clothes | 56 | Wears Kurdish and formal clothes | 100 | Wears Kurdish and formal clothes | 80 |
| Q15 | Which of the following characteristics are found in Hawleri | All (Hospitality, Charity, Honesty, Integrity, Faithful) | 89 | All (Hospitality, Charity, Honesty, Integrity, Faithful) | 50 | All (Hospitality, Charity, Honesty, Integrity, Faithful) | 64 |
| Q16 | If you are given the opportunity to live, where would you stay? | In Erbi | 100 | In Erbi | 100 | In Erbi | 84 |
| Q17 | In historical terms, tens of years ago Erbil | Was a Turkmani city | 67 | Other ethnicities | 50 | Was a Kurdish city | 88 |
| Q18 | In old historical terms, thousands of years ago Erbil | Was a Turkmani city | 44 | Other ethnicities | 50 | Was a Kurdish city | 76 |
